# Mirror Syndrome in Monochorionic Twin Pregnancy with Acardiac Fetus

**DOI:** 10.1155/2018/1302041

**Published:** 2018-01-08

**Authors:** Luiz Felipe Lessa Ortiz, Julio Elito Júnior, Edward Araujo Júnior, Alberto Borges Peixoto, Nelson Sass, Antonio Fernandes Moron

**Affiliations:** ^1^Department of Obstetrics, Paulista School of Medicine, Federal University of São Paulo (EPM-UNIFESP), São Paulo, SP, Brazil; ^2^Mário Palmério University Hospital, University of Uberaba (UNIUBE), Uberaba, MG, Brazil

## Abstract

We report the case of a 20-year-old patient, primigravida, with twin monochorionic pregnancy, with a weight gain of 6 kg in one week and increased blood pressure. During the ultrasound diagnostic investigation, placental edema and hydrops were identified in both fetuses, which, in association with maternal anasarca and pressure control, constitute the triad for mirror syndrome, also known as triple edema. In addition to being hydropic, one of the twins was an acardiac fetus, which is a rare combination of events. Gestation was terminated at 22 weeks and five days because of high maternal risk. The patient progressed with clinical and laboratory improvement. Our study is relevant in that it documents an extremely rare case and discusses relevant aspects of the symptoms and diagnosis of mirror syndrome. It also systematically reviews the condition.

## 1. Introduction

Mirror syndrome, also known as triple edema syndrome or Ballantyne syndrome, is rare and its prevalence and etiopathogenesis are unknown. It presents highly varied clinical, maternal, and fetal manifestations: maternal cutaneous edema, acute pulmonary edema, preeclampsia, polyhydramnios, and fetal and placental hydrops. It was first described by John William Ballantyne in 1892 as a condition associated with hydropic fetus and Rhesus (Rh) alloimmunization [1]. However, nonimmunogenic causes were found to be associated with this syndrome: placental chorioangioma, Ebstein's anomaly, viral infections in pregnancy, aneurysmal malformation of the Galen vein, and other fetal malformations [2–8]. In monochorionic twin pregnancy, two conditions that may progress to fetal hydrops are twin-to-twin transfusion syndrome and acardiac twin, also known as twin reversed arterial perfusion (TRAP) sequence. TRAP sequence is a rare obstetric condition that occurs in 1% of monochorionic twin pregnancies and in 1 : 35,000 of all pregnancies [9, 10]. Such a pathological entity occurs because of an anomalous perfusion circuit in which the donor twin (the “pump” twin) perfuses the receptor twin (the “acardiac” twin) through vascular (usually arterioarterial) anastomoses, resulting in reverse flow of deoxygenated blood to the receptor. This retrograde pattern of blood flow leads to striking abnormalities in the morphological development of the recipient, that is, absence or scarce development of cardiac and other structures such as the head, thorax, and upper limbs. The condition may worsen with fetal hydrops because of the blood pumping overload in the “pump” twin and, consequently, secondary heart failure in the same twin. Fetal hydrops are the trigger for mirror syndrome.

## 2. Case Report

**Figure 1:**
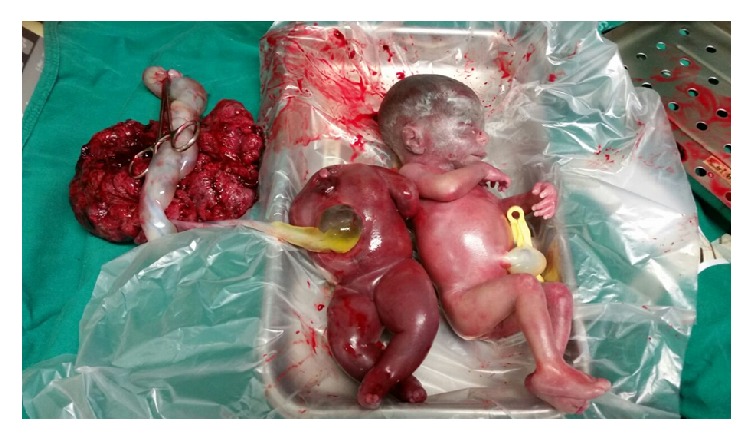
Hydropic placenta on the left; second acardiac and hydropic twin in the center; and first hydropic twin on the right.

**Figure 2:**
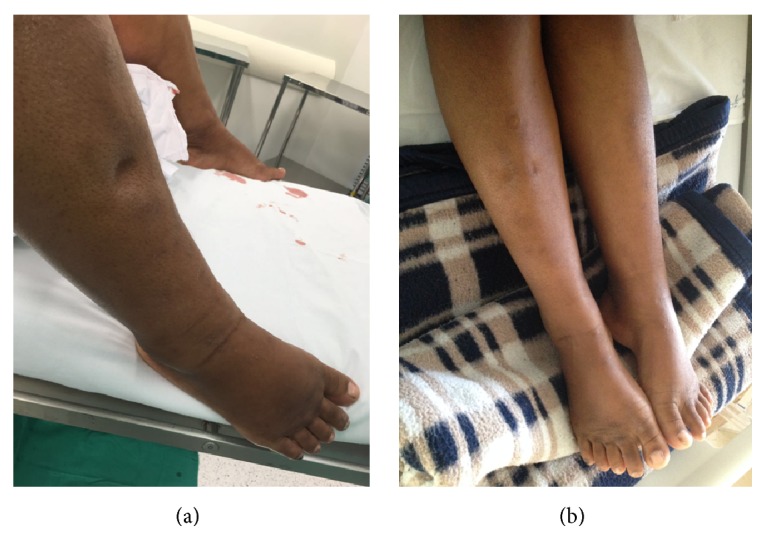
(a) Right lower limb showing edema on the day of labor induction with signs of Godet; (b) lower limbs on the second day after termination of gestation.

A 20-year-old woman, primigravida, with a gestational age of 22 weeks and three days was referred for high-risk prenatal care at the Department of Obstetrics, Paulista School of Medicine, Federal University of São Paulo, with a twin pregnancy, weight gain of 6 kg in the week prior, and increased blood pressure. Because 1 g/day of methyldopa was administered for two weeks before admission, she was asymptomatic at the time of admission, and she had a persistent blood pressure of 160/120 mmHg and edema in the lower limbs 4+/4+. She was referred for hospital admission. During examination, an obstetric ultrasound revealed that both the fetuses were hydropic and one of the twins had complex disruptive phenomena, without characterization of the cephalic pole and signs suggestive of TRAP sequence. Laboratory analysis showed 12 g of proteinuria in urine, microcytic/hypochromic anemia, and normal hepatic and renal function. Mirror syndrome, also known as triple edema or Ballantyne's syndrome, was diagnosed because of the presence of hemodilution, uncontrolled blood pressure level, significant maternal edema, and fetal hydrops. Due to severe preeclampsia, magnesium sulfate was administered by the Zuspan regimen but was suspended because of the risk of hypermagnesemia following the patient's evolution with oliguria and acute pulmonary edema. Because of the maternal risk and reserved fetal prognosis, gestation was terminated at 22 weeks and five days after discussion with the patient and family. Labor was induced by vaginal administration of misoprostol in accordance with the International Federation of Gynecology and Obstetrics (FIGO) protocol. Both the fetuses, birthed by vaginal delivery, were stillborn, with the first twin weighing 600 g, the acardiac twin weighing 375 g, and the placenta weighing 450 g (Figure 1). Postpartum recovery occurred in an Intensive Care Unit, with adequate blood pressure level after the administration of antihypertensives (hydralazine, methyldopa, and hydrochlorothiazide), with a significant reduction in edema, diuresis of 8000 ml within two days of furosemide therapy, and adequate control of serum potassium levels. The patient was scheduled for hospital discharge after improvement in laboratory test results and clinical symptoms upon the administration of a fourth antihypertensive, amlodipine, and puerperal follow-up for six days after gestational termination (Figure 2).

## 3. Discussion

The likelihood of acardiac fetus and mirror syndrome occurring concomitantly is of the order of 1/175 million; that is, this phenomenon is an extremely rare event. The probability of this event can be calculated by multiplying the incidences of both conditions. Mirror syndrome is a condition that occurs during pregnancy and has low prevalence. Given its rarity, there is a lack of clinical awareness about this condition and because of which it may go undiagnosed. This condition was first described by Ballantyne in 1892 and O'Driscoll in 1956, in association with severe cases of alloimmunization of the Rhesus (Rh) blood group and hydropic fetuses [11]. Although the pathophysiology of the syndrome is still unclear, it should be noted that studies show a possible correlation between angiogenic and antiangiogenic factors, studied more exhaustively in preeclampsia. When performing serial dosage of these factors (SFlt1, sEng, and P1GF), Espinoza et al. [12] concluded that there was an increase in antiangiogenic factors (sFlt 1 and sEng) and low concentration of P1GF (angiogenic), demonstrating a correlation between trophoblast damage with the release of antiangiogenic factors into the maternal blood circulation and endothelial dysfunction, resulting in elevation of pressure levels and preeclampsia. Despite hypotheses that aim to elucidate the pathophysiology of mirror syndrome, further studies are needed. In the medical literature, mirror syndrome is associated with immunological and nonimmunological causes. According to a systematic review of Ballantyne's syndrome, a series of 56 case studies described by Braun et al. immunological causes (mainly Rh alloimmunization) were the most prevalent, occurring in 15 cases. Other causes associated with the onset of the syndrome in the case studies included multiple pregnancies (10 cases); viral infections such as Coxsackie B, cytomegalovirus, and parvovirus B19 (nine cases); tumors and congenital anomalies such as placental chorioangioma, fetal aortic stenosis, Galen's vein aneurysm, cystic hygroma in the fetal neck, and fetal sacrococcygeal teratoma, among other unknown causes. In that same study, the symptoms of maternal edema and weight gain were the most common (89.3%), followed by increased blood pressure (60.7%), mild anemia and hemodilution (46.4%), proteinuria (42.9%), oliguria (16.1%), and acute pulmonary edema (21.4%), all of which were present in the patient's medical chart. Symptoms such as serum uric/creatinine acid elevation (25%), headache, and visual disturbances (14.3%) were absent [13]. Because of the occurrence of mirror syndrome at an early gestational age with important implications on maternal morbimortality, the medical literature shows that termination of gestation is the most appropriate way to protect maternal health even when faced with prematurity. When intrauterine fetal death occurs, the conduct may be expectant because of the possibility of improvement in maternal symptomatology [13]. In our case, maternal symptoms stopped six days after gestation was terminated. In this case, the medical decision to terminate gestation was of paramount importance for the sake of maternal life considering the severity of clinical symptoms, the early gestational age at which the symptoms occurred, and the inviability of the fetuses due to their precocious gestational age. The patient immensely benefitted from intensive care follow-up after the termination of gestation and showed rapid improvement.
